# Interrogating the transmission dynamics of *Trypanosoma cruzi* (Trypanosomatida, Trypanosomatidae) by *Triatoma venosa* (Hemiptera: Reduviidae) after the elimination of vector transmission by *Rhodnius prolixus* in Boyacá eastern Colombia

**DOI:** 10.3389/fcimb.2022.998202

**Published:** 2022-10-06

**Authors:** Manuel Medina, Sara Zuluaga, María Fernanda Martínez, Juan Carlos Bermúdez, Carolina Hernández, Virgilio Beltrán, Natalia Velásquez-Ortiz, Marina Muñoz, Juan David Ramírez, Omar Triana, Omar Cantillo-Barraza

**Affiliations:** ^1^ Programa de Control de Vectores, Secretaría de Salud Departamental, Tunja, Colombia; ^2^ Grupo Biología y Control de Enfermedades Infecciosas (BCEI), Universidad de Antioquia, Medellín, Colombia; ^3^ Centro de Investigaciones en Microbiología y Biotecnología – UR (CIMBIUR), Facultad de Ciencias Naturales, Universidad del Rosario, Bogotá, Colombia; ^4^ Molecular Microbiology Laboratory, Department of Pathology, Molecular and Cell-based Medicine, Icahn School of Medicine at Mount Sinai, New York, NY, United States

**Keywords:** Triatoma venosa, Rhodnius prolixus, Chagas disease, Triatomines, Secondary vectors, Colombia

## Abstract

Chagas disease (CD) is a parasitic zoonosis (*Trypanosoma cruzi)* that is endemic in Colombia. Vector control of *Rhodnius prolixus*, the main domestic *T. cruzi* vector, has been achieved in a large part of the area with historically vector transmission of CD. It is necessary to understand the ecological behavior characteristics of local native vectors to ensure sustainability of the vector control programs. To evaluate the long-term success of a recent vector control campaign in the Boyacá department (Colombia), we used a combined strategy of entomological surveillance with co-existing canine surveillance from ten rural villages within six municipalities of the Tenza valley region (Boyacá, Colombia): Chinavita, Garagoa, Guateque, Somondoco, Sutatenza and Tenza, with historical reports of *R. prolixus* and secondary vectors. Collected triatomines and canine whole blood were analyzed for *T. cruzi* infection and genotyping. Triatomine bugs specimens were evaluated for blood meal source. Canine serology was performed using two distinct antibody assays. In total, 101 *Triatoma venosa* were collected by active search in domestic and peridomestic habitats. A natural infection prevalence of 13.9% (14/101) and four feeding sources were identified: human, dog, rat, and hen. A frequency infection of 46.5% (40/87) was observed from two independent serological tests and *T. cruzi* DNA was detected in 14 dogs (16.4%). Only TcI_sylvatic_ DTU was detected. The results suggest that *T. venosa* present eco-epidemiological characteristics to maintain the transmission of *T. cruzi* in Tenza valley. This species has reinfested the intervened households and it has an active role in domestic and peridomestic transmission of *T. cruzi* due to their infection rates and feeding behavior. Therefore, this species should be considered as epidemiologically relevant for vector control strategies. Moreover, there is a need for human serological studies to have a close up of risk they are exposed to.

## Graphical Abstract

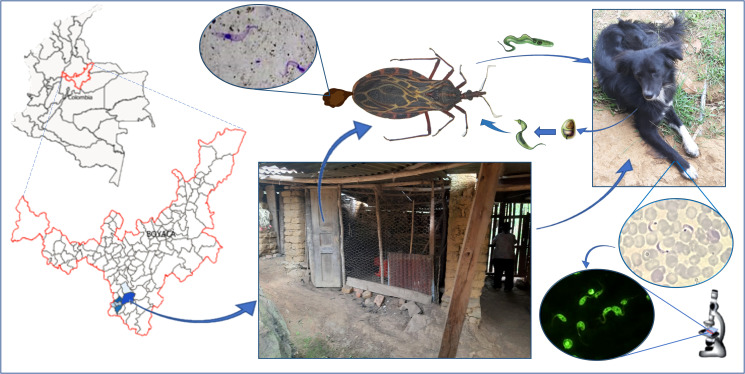

## Introduction

Chagas disease (CD) is an endemic zoonosis caused by *Trypanosoma cruzi.* This parasite is usually transmitted to vertebrate hosts by infected triatomine insects, commonly known as “the kissing bug” (Chagas, 1909). Triatome species of the genera *Triatoma* and *Rhodnius* are the most epidemiologically relevant for the transmission of *T. cruzi* in the Americas (WHO, 2007). At an epidemiological level, it is estimated that more than 6 million people are infected and approximately 12,000 dies annually (WHO, 2015). The control of the disease is focused on the elimination of house-infesting vectors, such as *Triatoma infestans, Triatoma dimidiata* and *Rhodnius prolixus*. Therefore, some international initiatives have been implemented INCOSUR (Initiative of the Southern Cone Countries), IPCAM (The Initiative of the Central American Countries and Mexico) and IPA (Initiative of the Andean Countries). Hence, contributing to the interruption of the transmission of *T. cruzi* in Latin America (Rojas de Arias et al., 2021).

Despite the success of these initiatives in reducing the number of cases of the disease, the parasite transmission by native triatomine vectors remains as the cause of thousands of new infections per year (Cardinal et al., 2007; Guhl et al., 2009; Cantillo-Barraza et al., 2014; Hernandez et al., 2016; Gorla and Noireau, 2017; Tovar Acero et al., 2017; Velásquez-Ortiz et al., 2022). Some countries certified by the PAHO-WHO as free from *T. cruzi* transmission have reported reinfestation events by native triatomine species, for instance *T. infestans* at the Chaco region and some other species such as *Triatoma brasiliensis, Triatoma pseudomaculata, Triatoma sordida* and *Panstrongylus megistus*, which maintain the transmission cycle of the parasite (Gurtler et al., 2007; Samuels et al., 2013; Espinoza et al., 2014). A similar situation has been described in countries part of the IPCAM, where *T. dimidiata* became the main domestic vector in areas once infested by *R. prolixus* (Hashimoto et al., 2006).

Colombia as part of the Initiative of the Andean Countries set up goals to achieve the interruption of *T. cruzi* transmission by *R. prolixus*, which is associated with domestic transmission (Guhl, 2007; WHO, 2007). After extensive control efforts across the region, 67 municipalities (half of them considered at high risk of vector-borne CD) were certified by PAHO-WHO as free of transmission by intradomestic *R. prolixus* (Ministerio de Salud, 2020). However, in Colombia there are 26 triatomine species (Guhl et al., 2007) of which 16 are known to be infected with *T. cruzi* (Guhl et al., 2007; Guhl and Ramirez, 2013; Cantillo-Barraza et al., 2015; Hernandez et al., 2016; Velásquez-Ortiz et al., 2022). *Triatoma venosa* is one of the species with the highest rate of domestic intrusion and colonization in Colombia (Guhl et al., 2007; Parra et al. 2015b). This species belongs to the *Triatoma dispar* lineage complex geographically restricted to Mesoamerica-Andes and composed by secondary and sylvatic vectors such as *Triatoma boliviana, Triatoma carrioni, Triatoma nigromaculata* and *T. dispar* (Monteiro et al., 2018). The eco-epidemiological study of this complex has been considered a priority for IPA (Initiatives of the Andean Countries) countries (Guhl et al., 2007; Coura et al., 2014; Rojas de Arias et al., 2021).

The enzootic transmission of CD in Colombia is associated with cultural and economic activities that can bring people closer to the enzootic cycle (Cantillo-Barraza et al., 2014; Villamil-Gomez et al., 2017; Zuleta-Duenas et al., 2017). However, the intrusion of species such as *Rhodnius pallescens* and *Panstrongylus geniculatus* attracted to the households by light or accidental food contamination by feces, are the most common ways of infection with sylvatic *T. cruzi* populations (Hernandez et al., 2016; Caicedo-Garzón et al., 2019; Cantillo-Barraza et al., 2021). Furthermore, the household-invading and colonizing triatomines *Triatoma maculata* and *T. dimidiata* bring enzootic and domestic transmission to households due to their interaction with reservoir mammals such as opossum, rodents, cats and dogs (Cantillo-Barraza et al., 2015; Hernandez et al., 2016; Cantillo-Barraza et al., 2020a).

Boyacá department has 24 municipalities recently certified by PAHO-WHO as free of *T. cruzi* transmission through the elimination of intradomiciliary *R. prolixus* (Cantillo-Barraza et al., 2021; Velásquez-Ortiz et al., 2022). However, recent evidence suggests that *T. cruzi* transmission risk maintained by other triatomine species, considering they can take advantage of the available niche, following the *R. prolixus* elimination (Cantillo-Barraza et al., 2020a; Cantillo-Barraza et al., 2021). The Tenza Valley, located in the southwest of the department, is a subregion Included in the elimination initiative (Corredor et al., 1990; Guhl et al., 2007). The chemical intervention for vector control performed in these municipalities developed by the Boyacá Health Department Service (BDHS) resulted in the elimination of *R. prolixus*. However, entomological surveillance between 2017 and 2018 following the chemical intervention showed the presence *T. venosa* inside the households (Cantillo-Barraza et al., 2021).

Understanding the epidemiological relevance of secondary native vectors in areas where primary vectors were eliminated is crucial to minimize the risk of transmission (Rojas de Arias et al., 2021). Several evidence showed that *T. dimidiata* is now the most important vector in this department (Cantillo-Barraza et al., 2021; Velásquez-Ortiz et al., 2022). However, the potential role of *T. venosa* in *T. cruzi* transmission is not yet clear (Vargas et al., 2006; Cantillo-Barraza et al., 2021). Therefore, this study aimed to describe the ecological characteristics of *T. cruzi* transmission in areas with reinfestation by *T. venosa* to estimate the potential risk of this triatomine species in the resurgence of CD transmission.

## Materials and methods

### Study area

**Figure 1:**
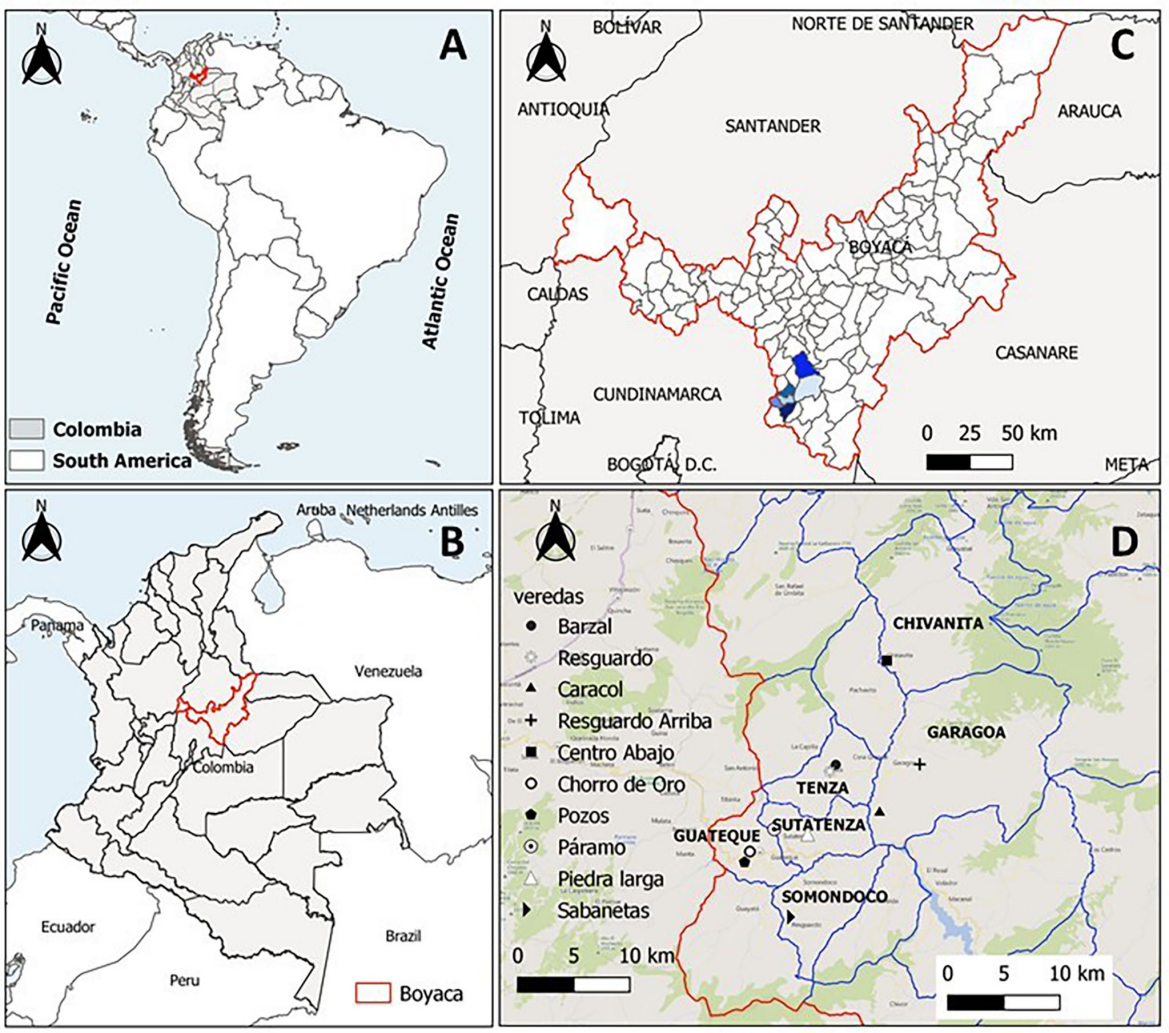
Geographical distribution of the samples in the study area and collection sites. **(A)** Map of south America highlighting Colombia, **(B)** Map of Colombia highlighting Boyacá, **(C)** Map of Boyacá highlighting the study area and **(D)** Municipalities of Valle de Tenza and villages evaluated.

This study was conducted between March and August of 2021 in six municipalities within the Tenza Valley subregion in Boyacá Department: Chinavita (5°29′31″N, 73°29′12″W), Garagoa (5°4′56.83″N, 73°21′54.17″W), Guateque (5°0′22″N, 73°28′17.12″W), Somondoco (4°59′8.75″N, 73°25′58.87″W), Sutatenza (5°1′´19.59″N, 73°27′11.22″W) and Tenza (5°4′38.32″N, 73°25′17.31″W) (**Figure 1**). This subregion comprises a Holdridge life zone classified as subtropical humid forest with an average annual rainfall of 1000 to 1500 mm and annual average temperature of 20°C. Rainy seasons are bimodal extending from April to June and August to November.

The municipalities of Chinavita, Garagoa and Sutatenza were certified free of *R. prolixus* intradomiciliary *T. cruzi* transmission in 2017 by PAHO, while Guateque, Somondoco and Tenza will be evaluated for certification in 2022 by PAHO. From each municipality, villages with household with *T. venosa* infestation reports after chemical-intervention for elimination of *R. prolixus* were selected for evaluation (**Table 1**) (Cantillo-Barraza et al., 2021). In addition, Triatomine Collection Stations (PITs-acronym in Spanish) for regular community surveillance activities were installed at each study location, as described by Cantillo-Barraza (Cantillo-Barraza et al., 2021).

**Table 1:** Summary of entomological information and *T. cruzi* infection in *T. venosa* specimens.

Municipality	Village	Houses evaluated by village					Number Triatomines collected (nymphs/ adults)	% Infection of triatomines
		n	Infestation index (%)	Colonization index (%)	Number Triatomines in domicilie	Number Triatomines in peridomicilie		
**Chinavita**	Centro Abajo	72	1.38	100	2	1	3 (2/1)	0
**Garagoa**	Caracol	130	0	0	----	----	---	---
	Resguardo Arriba	97	4.2	25	2	2	4 (1/3)	0
**Guateque**	Chorro de Oro	233	2.1	50	6	13	19 (5/14)	26
	Pozos	65	21.5	7.1	3	20	23 (1/22)	13
**Somondoco**	Sabanetas	70	5.7	80	5	9	14 (4/10)	28.6
**Susatenza**	Paramo	405	0.74	0	0	1	1 (0/1)	0
	Piedra Larga	177	0	0	----	---	---	---
**Tenza**	Barzal	212	3.3	100	17	2	19 (15/4)	10.5
	Resguardo	238	1.2	33	18	0	18 (17/1)	0
**Total**		1602	2.6		53	48	101 (45/56)	13.9

### Triatomine collection and processing

From March to August 2021, three entomological surveys were performed at each study location. All procedures were carried out by BDHS technicians, following the National Protocols of Entomological Surveillance (Ministerio de Salud, 2020). Triatomines were searched in indoors (intradomicile) and outdoors (peridomicile) niches for 30 min following the steps below: a flashlight was used to see into wall cracks and crevices, behind wall-mounted picture frames, behind furniture, inside closets and particularly under bedding material. Triatomines were transported to the laboratory, registered and identified using taxonomic keys (Lent and Wygodzinsky, 1979).

All triatomines collected were evaluated for *T. cruzi* infection using parasitological and molecular methods at the Universidad de Antioquia, Medellín, Colombia. Feces were obtained by abdominal compression, diluted in 300 μL of sterile PBS (pH: 7.2) and used for *T. cruzi* DNA extraction. Genomic DNA was extracted from 200 µL of feces using the DNeasy Blood & Tissue Kit, (Qiagen, Germantown, MD, USA) following manufacturer’s instructions.

### Dog sampling

During each entomological survey, dogs were selected from infested households in houses with sighting reports by householders in the last year and houses that reported infestation events (Cantillo-Barraza et al., 2021). Inclusion criteria for these mammals were as follows: (I) to have born and raised in the study area, (II) having a recognizable owner and (III) an informed consent from their owners. For each animal, two 5mL radial vein blood samples were collected, using Serum and K3-EDTA vacutainers and stored at 4°C until processed. For serum processing, samples were centrifuged at 5000 g for 10 min and extracted serum was stored at -20°C until diagnostic assays were performed. Genomic DNA was extracted from 200 µL of K3-EDTA vacutainer collected whole blood using Dneasy Blood & Tissue Kit, (Qiagen^®^) according to the manufacturer’s instructions. Total DNA was diluted with 100 µL elution buffer and stored at -20°C until molecular diagnosis.

### Molecular detection of *T. cruzi* infection

All collected *T. venosa* and domestics dogs were screened for *T. cruzi* using a conventional PCR targeting satellite DNA (Moser et al., 1989). The PCR was performed in a final volume of 25µL containing 40-50 ng of genomic DNA, 1X of buffer, 0.04 mM of dNTP, 1.5 mM MgCl_2_, 0.4 µM of each primer (TCZ1 and TCZ2), and 0.05 U of Taq DNA polymerase (Invitrogen, Carlsbad, CA, USA). The thermal cycling conditions were as follows: pre-heating at 95°C for 15 min, 40 cycles at 95°C for 10 s, 55°C for 15 s, and 72°C for 10 s in a thermal cycler. Positive *T. cruzi* samples were analyzed for molecular discrimination of *T. cruzi* discrete typing units (DTUs) based on the amplification of spliced leader intergenic region (SL-IR) gene using the primers TCC, TC1 and TC2, as previously reported (Hernández et al., 2016). The PCR was performed in a final volume of 25µL containing 40-50ng of genomic DNA, 1X of buffer, 0.25 mM of dNTP, 2 mM MgCl_2_, 0.4µM of each primer, and 0.05 U of Taq DNA polymerase (Invitrogen). Thermal cycling conditions were as follows: pre-heating at 94°C for 5 min, 35 cycles at 94°C for 30 s, 55°C for 30 s, and 72°C for 45 s in a thermal cycler, and a final extension at 72°C for 5 min. Amplification products were run on a 1.5% agarose gel stained by ethidium bromide and visualized under UV light. For direct sequencing of SL-IR region, PCR products were purified and sequenced using Sanger methodology through Macrogen Sequencing Service, (Seoul, South Korea).

For TcI genotype identification, the microsatellite motif of the spliced leader gene (positions ranking between ~14 to ~40) was omitted as suggested (Tomasini et al., 2011). All nucleotide sequences were aligned using CLUSTALW as implemented in BioEdit v.7.1.9 Hall 1999. Then, the highest sequence nucleotide identity values were calculated in BioEdit v.7.1.9 Hall 1999. Based on SL-RI sequence optimal global pairwise alignments against reference strains reported for Colombia (Cura et al., 2010).

### Blood-meal sources in triatomines

Triatomine bloodmeal sources were identified by vertebrate 12S rRNA gene PCR targets, and were obtained through amplification of a 215 bp fragment using primers L1085 (5′-CCCAAACTGGGATTAGATACCC-3′) and H1259 (5′-GTTTGCTGAAGATGGCGGTA-3′) (Dumonteil et al., 2007) Electrophoresis was performed on a 1.5% agarose gel stained with SYBR Safe and visualized under UV light into a molecular imager^®^ Gel DOCTM XR+ with Image LabTM software (Bio-Rad Laboratories Inc, California, USA). The resulting sequences in both directions were edited in MEGA X software, assembled and manually checked in all base changes according to the quality of peaks (height, not overlapping and evenly spaced) prior to be submitted to BLASTn in NCBI (https://blast.ncbi.nlm.nih.gov) for similarity search defining each species with percent identity higher than 98% and e-value close to 0,00.

### Serological canine diagnostic testing

Detection of anti-*T. cruzi* antibodies (IgG) in dogs were conducted using an Enzyme-Linked Immunosorbent Assay (ELISA) and an Indirect Immunofluorescence Antibody Test (IFAT). For both techniques, the antigen was prepared from harvested epimastigotes of *T. cruzi* Colombian strains (I.RHO/CO/00/CAS-15.CAS; I. TRI/CO/03/MG-8.MAG), previously characterized as TcI (Cantillo-Barraza et al., 2015). For ELISA, a whole lysate extracted from epimastigotes was used as antigen, while IFAT utilized complete epimastigotes fixed in 1% formaldehyde (Cantillo-Barraza et al., 2015). The cut-off was determined as optical absorbance ≥ 0.200 (mean ± SD of negative control) for ELISA and sera dilution of ≥ 1/40 for IFAT as reported elsewhere (da Xavier et al., 2012). Animals were defined as positive when samples were reactive to both tests, which have a 100% sensitivity and 98.7% specificity reported for the ELISA and IFAT (Bio-Manguinhos, FIOCRUZ, Rio de Janeiro, Brazil) (Xavier et al., 2012).

### Geospatial analysis

Base maps were extracted from the GADM database (www.gadm.org). Google Earth v.2.5 was used to determine the coordinates for all municipalities where triatomine insects and domestic dog samples were collected. The individual study location coordinates were captured using a hand-held GPD (Global Positioning System). Coordinates were recorded in the WGS 84 Datum (Macomber 1984) geodetic coordinate system.

### Ethics statement for animal evaluations

All animals were handled in strict accordance with good animal welfare as defined by the Colombian code of practice for the care and use of animals for scientific purpose, established by law 84 of 1989. Ethical approval (Act No. 113 of 2017) for analyzing animal specimens was obtained from the Animal Ethics Committee at the Universidad de Antioquia.

## Results

### *T. venosa* entomological survey, *T. cruzi* infection rate, and blood meal sources

A total of 101 *T. venosa* were collected in eight of ten villages evaluated in the six investigated municipalities (**Table 1**). The 52% of triatomines were collected in domestic and 48% in peridomestic habitats. Most of the specimen were adults (55%) and the remaining 45% were nymphal instars. Among captured *T. venosa* specimens, 13.9% (n=14/101) were positive by PCR. *T. cruzi* prevalence was highest in Sabanetas (28.6%), Chorro de Oro (26%) and Pozos (13%) (**Table 1**). Lastly, only TcI_sylvatic_ DTU was found in positive *T. venosa* (**Table 2**).

**Table 2:** Genotypes of TcI found in infected dogs and *T. venosa* in the study area.

Municipality	Puppy	Young	Adults	Geriatric	Genotype of *T. cruzi* in infected dogs	Genotype of *T. cruzi* in *T. venosa*
**Garagoa**	0	2	1	2	TcI sylvatic	–
**Guateque**	1	2	0	1	TcI sylvatic	TcI sylvatic
**Somondoco**	–	–	–	–	–	TcI sylvatic
**Tenza**	0	1	2	1	TcI sylvatic	TcI sylvatic

**Figure 2:**
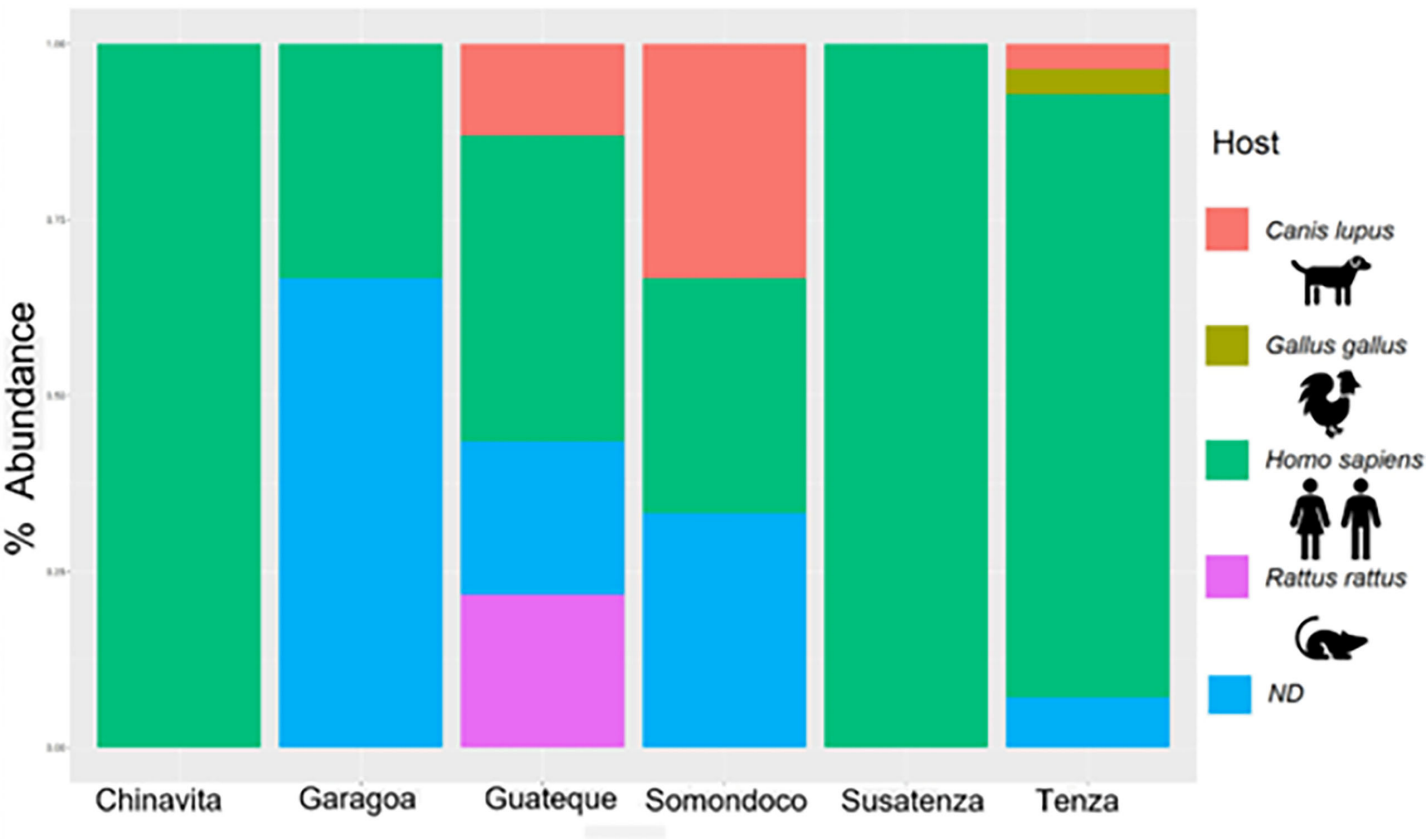
Summary of feeding profiles of *T. venosa* specimens per municipality. The barplot shows top 4 blood meals *T. venosa* fed on.

Four blood meal sources were found among the 61 *T. venosa* specimens with sufficient genetic material for analysis. The most frequent were *Homo sapiens* (human) 65% (40/61), *Canis lupus familiaris* (domestic dogs) 10% (6/61), *Rattus rattus* 8% (5/61), and *Gallus gallus* 2% (1/61). In 15% of *T. venosa* with sufficient DNA, bloodmeal source did not match any BLAST dataset sequences. Geospatially, all Chinavita, Garagoa and Sutatenza collected *T. venosa* were human-only blood feeders, whereas Guateque, Tenza and Somondoco collected *T. venosa* exhibited multiple bloodmeal sources (**Figure 2**). An interesting find was that nymphal states were only found inside the dwellings and the 90% of these nymphs fed of humans, while adults fed on *R. rattus* and *G. gallus*.

### *Trypanosoma cruzi* infection in domestics dogs

A total of 86 domestics dogs with a mean age of 4.1 ± 0.7 years (range of 6 months to 15 years) were collected in 69 houses within the six municipalities (**Table 3**). Chagas seropositivity was detected in 44.1% (n= 38/86) and *T. cruzi* DNA was present in 36.4% (n= 14/38) of seropositive dogs (**Table 1**). In five municipalities, seropositive puppies and youthful dogs were identified, where Somondoco was the exception. *Trypanosoma cruzi* PCR positive dogs were distributed in three municipalities: Garagoa, Guateque and Tenza. Furthermore, in Guateque and Tenza *T. cruzi* PCR positive puppies were found. Along with infected *T. venosa* genotype findings, *T. cruzi* PCR positive dogs also only exhibited TcI _sylvatic_ DTU (**Table 2**).

**Table 3:** *T. cruzi* infection and serological data of dogs per age, sex and village.

Variable	Dogs enrolled n=86 (%)	Seropositive n=38 (%)	Seronegative n=48 (%)	PCR positives n=14 (%)
**Age (Years)**	4.1 ± 0.7			
**Puppy (3m to <1y)**	5 (5.8)	3 (7.9)	2 (4.2)	2 (14.3)
**Young (1> to 4 y)**	47 (54.6)	19 (50)	28 (58.3)	5 (35.7)
**Adults (>4 y to 7y)**	15 (17.4)	7 (18.4)	8 (16.6)	3 (21.4)
**Geriatric (>7y)**	19 (22)	9 (23.6)	10 (20.8)	4 (28.6)
**Sex**				
**Male**	51(60)	26 (68.4)	25 (52)	11 (78.6)
**Female**	35(40)	14 (36.8)	21 (43.7)	3 (21.4)
**Villages**				
**Centro Abajo**	14 (16.3)	4 (10.5)	10 (20.8)	0 (0)
**Caracol**	11 (12.6)	6 (15.8)	5 (10.4)	4 (28.6)
**Resguardo Arriba**	5 (5.7)	1 (2.6)	4 (8.3)	1 (7.1)
**Chorro de Oro**	10 (11.5)	6 (15.7)	4 (8.3)	3 (21.4)
**Pozos**	9 (10.4)	6 (17.7)	3 (6.2)	1 (7.1)
**Sabanetas**	5 (5.7)	0 (0)	5 (10.4)	0 (0)
**Páramo**	7 (8.1)	1 (2.6)	6 (12.5)	0 (0)
**Piedra Larga**	7 (8.1)	4 (10.5)	3 (6.2)	0 (0)
**Barzal**	10 (12.3)	7 (18.4)	3 (6.2)	3 (21.4)
**Resguardo**	8 (9.3)	3 (7.9)	5 (10.4)	2 (14.2)

## Discussion

Vector control initiatives from Latin America demand entomological surveillance based on understanding the ecological, genetic and behavioral features of native vectors (Monteiro et al., 2013; Rojas de Arias et al., 2021). In Colombia, *T. dimidiata* and *T. venosa* are secondary vectors of *T. cruzi* present in the domestic-peridomestic niches following the elimination of *R. prolixus* (Cantillo-Barraza et al., 2020a; Velásquez-Ortiz et al., 2022). Some authors highlight the eco-epidemiological relevance of *T. dimidiata* and the need to include them in the vector control programs (Gómez-Palacio et al., 2013; Parra-Henao et al., 2015a; Quirós-Gómez et al., 2017; Cantillo-Barraza et al., 2020a; Cantillo-Barraza et al., 2021; Velásquez-Ortiz et al., 2022). However, the eco-epidemiological features, feeding habits, natural infection rates and transmission dynamics for *T. venosa* are barely known (Vargas et al., 2006; Cantillo-Barraza et al., 2021). Here, we suggest that *T. venosa* is a re-invasive, re-infesting and colonizing triatomine species with relevance for the *T. cruzi* transmission across domestic and peridomestic areas in the Boyacá department, where these species maintain an ecological relationship with local dogs, therefore contributing for peridomestic *T. cruzi* maintenance.

The Initiative of the Andean Countries (IPA) in Colombia has shown advances in the elimination of domiciled primary species and improve the understanding of the biology and ecology of native species associated with humans as *T. dimidiata*, but little progress has been made in the study of the eco-epidemiological characteristics of others species reported for a long time inside the dwellings such as *T. venosa* (Corredor et al., 1990; Molina et al., 2000; Guhl et al., 2009; Parra-Henao et al 2015b; Costa et al., 2021; Rojas de Arias et al., 2021; Cantillo-Barraza et al., 2021; Velasquez-Ortiz et al., 2022). The scarce information about the ecology of this species could suggests a little epidemiological relevance in Chagas disease transmission (Abad-Franch and Monteiro, 2007; Guhl et al., 2007; Guhl and Ramirez, 2013). In Ecuador, *T. venosa* is a wild arboreal species with distribution in montane forest on both sides of the Andes and without potential risk for *T. cruzi* transmission (Abad-Franch et al., 2001; Vaca-Moyano et al., 2017; Abad-Franch and Gurgel-Gonçalves, 2021). In Colombia, *T. venosa* has been reported in households of 87 municipalities in eight departments and its sylvatic habitat remains unknown (Corredor et al., 1990; Molina et al., 2000; Parra-Henao et al 2015b). However, its sympatric distribution with vectors of greater epidemiological importance such as *R. prolixus*, *T. dimidiata* and *P. geniculatus* has concealed on its true importance in the transmission of *T. cruzi* (Corredor et al., 1990; Molina et al., 2000; Guhl et al., 2007; Parra-Henao et al 2015b; Cantillo-Barraza et al., 2021).The present study showed reinfestation of 2.6% of dwellings in the study area (**Table 1**). In addition, 100% of the nymphs collected were found inside the dwellings, suggesting re-colonization of this species into domestic habitats after chemical intervention. These results, together with other studies conducted in the area by our working group since 2017 where infestation and colonization indices were lower, demonstrate that the re-infestation and colonization processes carried out by *T. venosa* are sustained and increasing (Cantillo-Barraza et al., 2021). Similar eco-epidemiological situations regarding the increase in entomological indices have been described for *T. dimidiata* the major intradomiciliary vector of *T. cruzi* in Colombia (Cantillo-Barraza et al., 2021; Velásquez-Ortiz et al., 2022), as well as, for *T. brasiliensis, T. pseudomaculata, T. sordida* and *P. megistus* in Southern Cone countries (Rojas de Arias et al., 2021).

One of the most important results we obtained was the frequency of natural infection of *T. venosa* in Colombia. The information available for this species shows certain gaps on its epidemiological relevance. In Ecuador, the natural infection status of wild populations present in the country is unknown (Abad-Franch et al., 2001; Vaca-Moyano et al., 2017), while in Colombia the presence of DTU I, II and IV has been reported but the prevalence of the infection in domestic and peridomestic populations is not clear (Guhl et al., 2007; Guhl and Ramirez, 2013; Parra-Henao et al 2015b). We report an infection rate of 13.9% (14/101), which is the double than reported in a study conducted between 2017 and 2018 for the same department, suggesting that the potential risk by this species has been increasing (Cantillo-Barraza et al., 2021). However, *T. venosa* prevalence infection is lower than reported by other species of greater epidemiological relevance as *R. prolixus* (55.84) and *T. dimidiata* in Colombia (40% -70.31%) (Hernandez et al., 2016; Cantillo-Barraza et al., 2020a; Velazquez-Ortiz et al., 2022). On the other hand, similar prevalence values have been reported by other secondary species with an active role in the transmission cycle of *T. cruzi* following the primary vectors elimination, such as *T. brasiliensis* (2.8 – 15.8%), *T. pseudomaculata* (0 – 12.8%), *T. sordida* (0 – 3.75%) and *P. megistus* (0 – 11.6%) (Lima et al., 2012; Monteiro et al., 2013; Rojas de Arias et al., 2021).

Regarding the feeding behavior, we found that *T. venosa* fed from at least 4 different sources of blood including domestic and peridomestic animals (human, domestic dog, chicken and rat) (**Figure 2**), with *H. sapiens* blood as the most frequent. These ecological characteristics have been described for three principal triatomine genera implicated in *T. cruzi* transmission: *Panstrongylus, Rhodnius* and *Triatoma* (Carrasco et al., 2005; Cecere et al., 2016; Hernandez et al., 2016; Ocaña-Mayorga et al., 2021). Moreover, the ability of *T. venosa* to feed on species present in peridomestic ecotopes as *R. rattus* has also been reported (Arias-Giraldo et al., 2020). However, the results presented here show that *T. venosa* has a more generalist feeding behavior in which mammals are the main source of blood. This eclectic behavior has been described for other species with greater epidemiological relevance and domiciliation capacity such as *R. prolixus* (Pena-Garcia et al., 2014), *T. dimidiata* (Hernandez et al., 2016; Velásquez-Ortiz et al., 2022), *P. geniculatus* (Hernandez et al., 2016; Arias-Giraldo et al., 2020) and *T. maculata* (Cantillo-Barraza et al., 2015; Hernandez et al., 2016).

The presence of TcI _sylvatic_ found in this study and reports of infection with TcI _Dom_, support the active role of *T. venosa* in the domestic and peridomestic transmission of *T. cruzi* in the Tenza valley (Boyacá) (Cecere et al., 2004; Guhl and Ramirez, 2013; Leon et al., 2015; Cantillo-Barraza et al., 2021). The participation of *T. venosa* in scenarios where different parasite reservoirs and genotypes converge, as well as their closer contact with humans, implies *T. venosa* might represent a risk for *T. cruzi* transmission considering their mobility and blood intake from many domestic and synanthropic reservoirs, as described for *T. dimidiata* and *T. maculata* in other zones of Colombia (Cantillo- Barraza *et al.*, 2015*;* Hernandez et al., 2016 *;* Velásquez-Ortiz et al., 2022). Therefore, our results showed *T. venosa* should be considered as epidemiologically relevant and should be included in vector control strategies. Additionally, we highlight the urgence of serological studies of the residents to have a close up of the risk they are exposed to.

Furthermore, the role of domestic dogs in the CD epidemiology is different by region (Jansen and Roque, 2010). In south cone countries and Venezuela, infected dogs have been associated with a higher risk of human infection (Gurtler et al., 2007; Enriquez et al., 2013). A different scenario has been reported in Brazil, where dogs are considered to have little epidemiological importance (Jansen and Roque, 2010). These eco-epidemiological differences also have been observed in Colombia (Ramirez et al., 2013; Jaimes-Dueñez et al., 2017; Cantillo-Barraza et al., 2020b). The results of this work suggested that domestic dogs have an important role in the maintenance of *T. cruzi* peridomestic transmission cycle in the study area. We found *T. cruzi* seropositivity of 44.4% in domestic dogs and the presence of *T. cruzi*-DNA in the 36.9% of them. Also, dogs were the second most frequent blood source for *T. venosa* (Jansen and Roque, 2010). Domestic dogs likely constitute the link between the domestic and sylvatic environments, as reported in other Colombian regions (Ramirez et al., 2013; Jaimes-Duenez et al., 2017; Cantillo-Barraza et al., 2020b). Culturally in Colombia, dogs are used as housing protection and are permanently tied to the housing structure. However, in the interviews with our study communities, it was revealed that dogs are used to attack opossums that arrive at homes in search of solid waste or domestic birds to feed. It is possible that this situation contributed to the increase of TcI _sylvatic_ frequency we reported here (Cantillo-Barraza et al., 2020b).

Although the intradomestic vector elimination initiatives in Latin America are indisputable, some authors warn about the false sense of security generated after these public health goals have been achieved (Canals et al., 2021; Rojas de Arias et al., 2021). The detection of domestic and peridomestic transmission in areas certified as free from intradomiciliary transmission of *T. cruzi* should be a concern for vector control and public health agencies, considering *T. venosa* could be involved in the enzootic transmission into households, as described for *T. dimidiata* and *T. maculata* (Cantillo- Barraza et al., 2015 *;* Hernandez et al., 2016 *;* Velazquez-Ortiz et al., 2022). Therefore, our results highlight the need for a shift of these epidemiological scenarios throughout Latin America and adopt more sensitive tools that allow real-time identification of emerging foci (Cantillo-Barraza et al., 2015; Hernandez et al., 2016; Gysin et al., 2022). In Colombia, a primary deficiency is the lack of optimal infrastructure and trained personnel for Chagas disease molecular surveillance in departments and other decentralized entities responsible (WHO, 2007). Besides, due to the SARS-CoV-2 pandemic contingency, Colombia has formed a decentralized network of molecular biology laboratories that could be used for molecular surveillance of Chagas disease and other vector-borne diseases. Finally, our results highlight the emergence of *T. venosa* as a public health Chagas disease vector of concern that is actively colonizing domestic and peridomestic environments in the post eradication era of *R. prolixus*.

## Data availability statement

The original contributions presented in the study are included in the article/supplementary material. Further inquiries can be directed to the corresponding author.

## Ethics statement

The animal study was reviewed and approved by Ethical approval (Act No 113 of 2017) for analyzing animal specimens was obtained from the Animal Ethics Committee at the SIU- Universidad de Antioquia. Written informed consent was obtained from the owners for the participation of their animals in this study.

## Author contributions

Conceptualization: OCB, OT, MMe and JDR. Data curation: OCB, SZ, MMa, CH, MMu and VB. Formal Analysis: OCB, SZ, NV, CH, JDR and OT. Investigation: OCB, MMe, SZ, VB, JB, NV, MMu, JDR and OT. Funding acquisition: OCB, MMe, VB, JDR, MMu and OT. Writing-review & editing: MMe, JDR, OT and OCB. All authors contributed to the article and approved the submitted version.

## Funding

This study was carried out thanks to the agreement No. 2788 of 2021 signed between the Health Secretary of the Department of Boyacá, and the University of Antioquia (Biology and Control of Infectious Diseases Group, BCEI). To prof. Samanta Cristina da Chagas Xavier for Map elabortion.

## Conflict of interest

The authors declare that the research was conducted in the absence of any commercial or financial relationships that could be construed as a potential conflict of interest.

## Publisher’s note

All claims expressed in this article are solely those of the authors and do not necessarily represent those of their affiliated organizations, or those of the publisher, the editors and the reviewers. Any product that may be evaluated in this article, or claim that may be made by its manufacturer, is not guaranteed or endorsed by the publisher.
